# Machine Learning-Enabled Layer-Wise Melting Quality Recognition for Laser Powder Bed Fusion Process via In Situ Monitoring

**DOI:** 10.3390/ma19122463

**Published:** 2026-06-09

**Authors:** Yuan Liu, Bowei Zou, Zhizhou Zhang, Yongxing Zhang, Shiqing Huang

**Affiliations:** 1 School of Mechanics and Construction Engineering, Jinan University, Guangzhou 510632, China; ly600@jnu.edu.cn (Y.L.);; 2 Electric Power Dispatching and Control Center of Guangdong Power Grid Co., Ltd., Guangzhou 510600, China; zoubowei1996@163.com; 3 Laser Processing Research Laboratory, School of Engineering, The University of Manchester, Manchester M13 9PL, UK; 4 School of Electromechanical Engineering, Guangdong Polytechnic Normal University, Guangzhou 510665, China; yongxingzhang@gpnu.edu.cn; 5 College of Packaging Engineering, Jinan University, Zhuhai 519070, China

**Keywords:** laser powder bed fusion, in situ monitoring, machine learning, melting quality recognition, defects classification

## Abstract

Laser powder bed fusion (L-PBF) has emerged as a core metal additive manufacturing technology for high-end sectors, including aerospace and medical device manufacturing. However, melting anomalies that occur during fabrication accumulate layer by layer, leading to degraded surface quality and impaired mechanical performance of as-built components—a critical bottleneck limiting their large-scale industrial adoption. Accurate and robust layer-wise melting quality recognition remains a challenge due to the complex surface morphologies induced by such melting anomalies. This study presents a machine learning-enabled in situ monitoring approach for layer-wise melting quality identification in L-PBF. By systematically varying laser power and scanning speed, 24 parameter combinations were designed to fabricate specimens with three distinct melting states: over-melting (OM), lack of fusion (LOF), and normal melting. A high-resolution complementary meta–oxide–semiconductor (CMOS) camera was used to capture layer-wise surface images of the specimens, and following abnormal layer filtering and manual validation, a high-quality dataset comprising 5110 layer-wise images was constructed. Two mainstream machine learning approaches were systematically evaluated and optimized for melting quality classification: a support vector machine (SVM) model leveraging handcrafted gray-level co-occurrence matrix (GLCM) texture features achieved a classification accuracy of 96.77%, while a convolutional neural network (CNN) model with end-to-end feature learning directly from raw images attained a superior accuracy of 98.14%. In terms of computational efficiency, the CNN model exhibited a faster inference speed with a per-layer inference time of just 0.036 s, nearly half that of the SVM model (0.068 s per layer). Most critically, the CNN model completely eliminated fatal cross-class misclassification between OM and LOF—an error mode common in the SVM model that would trigger erroneous process corrective actions in practical industrial applications. The findings demonstrate that image-based machine learning provides a reliable technical foundation for intelligent in situ monitoring of the L-PBF process. With its high accuracy, strong robustness, and superior computational efficiency, the CNN model can effectively support on-site operational decision-making, reduce material and time losses, and enhance process stability in industrial settings, thus exhibiting significant potential for practical engineering deployment.

## 1. Introduction

Additive manufacturing (AM) has emerged as a transformative manufacturing technology widely deployed in high-end sectors including aerospace, biomedical devices, and automotive engineering, owing to its unparalleled capabilities in functional integration, lightweight structural optimization, near-net-shape fabrication of complex geometries, and mass customization of personalized products [1,2,3,4]. As one of the most mature and industrially validated metal AM technologies, laser powder bed fusion (L-PBF) employs a high-energy laser beam to selectively melt and consolidate metal powder beds layer-by-layer in accordance with digital 3D model data, enabling the fabrication of high-density metallic components with intricate architectures [5,6,7].

Despite these advantages, the widespread industrial uptake of L-PBF remains severely hindered by poor quality consistency of as-built parts and inadequate process repeatability across batches—core challenges that restrict its large-scale application in safety-critical fields. Typical process-induced defects, including residual stress accumulation, microcrack initiation, dimensional deviation, and interlayer bonding defects, can significantly degrade the critical mechanical performance of fabricated components, such as tensile strength, wear resistance, and fatigue life [8,9]. The final performance of L-PBF-fabricated parts is fundamentally governed by the melting quality of each individual layer during the build process: the dynamic in-chamber environment, including laser-induced fumes, spatter generation and redeposition, and layer-wise thermal accumulation, can directly alter the surface morphology of the as-melted layer. These interlayer disturbances accumulate progressively throughout the build cycle, ultimately leading to irreversible degradation of the mechanical properties and dimensional accuracy of the final component [10].

The L-PBF process is inherently a complex multi-physics coupling process, involving rapid heat transfer, melt pool fluid dynamics, and solid–liquid–gas phase transitions within an extremely short interaction time window. Under such highly dynamic conditions, melt pool stability is extremely sensitive to various perturbations: inappropriate process parameter selection, laser beam drift, and laser energy attenuation caused by fumes and spatter can all disrupt melt pool stability, leading to anomalous melting states and associated process defects [11,12,13]. In practice, L-PBF process instability is mainly manifested in two typical anomalous melting states: over-melting (OM) and lack of fusion (LOF). The OM is triggered by excessive laser energy input, which creates an overly vigorous melt pool accompanied by violent spatter generation and metal vapor plume emission; unremoved spatter particles redeposit on the as-melted surface, leading to increased surface roughness and local contamination.

In contrast, lack of fusion is caused by insufficient laser energy density, where metal powder cannot be completely melted and consolidated, resulting in poor wettability and fluidity of the molten metal, discontinuous melt tracks, balling defects, and deteriorated surface quality [14,15]. Without timely in situ detection and intervention, these anomalous melting states cause uneven powder spreading in subsequent layers, trigger progressive defect accumulation, and even lead to unexpected build failure, resulting in significant waste of raw materials, production time, and labor costs.

To address these challenges, in situ process monitoring of L-PBF has been widely recognized as the most promising solution to enable real-time quality control [16,17,18,19,20]. By capturing and analyzing process signals during the build cycle, in situ monitoring systems can identify the type and severity of melting anomalies in real time, providing critical decision support for operators to implement timely corrective actions or terminate defective builds early, thus minimizing production losses. Various sensing modalities have been investigated for L-PBF in situ monitoring, including acoustic emission, high-speed melt pool imaging, and machine vision-based layer-wise surface monitoring [21,22,23,24,25,26]. Among these, layer-wise surface imaging via machine vision has attracted extensive attention due to its non-intrusive nature, high spatial resolution, and direct correlation with as-built surface quality. For instance, Jiang et al. [27] developed a machine vision-based in situ monitoring system that captured layer-wise surface images and adopted deep learning to identify layer warpage defects; Zhao et al. [28] proposed an uncertainty-driven defect detection method for L-PBF powder bed images, which fused deep semantic features and shallow texture features to achieve high-precision segmentation of five typical powder spreading defects; Ma et al. [29] established a layer-wise image dataset of three typical melting states (OM, LOF, and normal melting) and developed a deep learning model for in situ melting quality recognition, which was further used for online process parameter optimization.

While these pioneering studies have validated the feasibility of machine learning (ML)-based layer-wise quality recognition for L-PBF, two critical research gaps remain to be addressed. First, most existing studies treat the ML classification model as a “black box”, with limited systematic investigation into how model architectures, hyperparameters, and feature engineering strategies affect recognition performance. This lack of interpretability provides little practical guidance for model selection and optimization in industrial L-PBF applications where process conditions and hardware configurations vary widely. Second, existing research primarily focuses on improving overall classification accuracy, with little attention paid to the type of misclassification; in particular, cross-class misclassification between OM and LOF—two physically opposite anomalies that require completely opposite corrective actions—can lead to catastrophic operational errors in industrial practice, yet this critical issue has been rarely discussed in existing literature.

To fill these research gaps, this study presents a systematic investigation of ML-driven layer-wise melting quality recognition for L-PBF in situ monitoring. First, a series of L-PBF experiments were conducted by systematically varying laser power and scanning speed to fabricate specimens with three typical melting states (OM, LOF, and normal melting), and a non-intrusive in situ monitoring platform integrated with a high-resolution CMOS camera was established to capture layer-wise surface images of the as-melted parts during the build process. A high-quality labeled dataset was then constructed after abnormal layer filtering and manual verification. Second, two representative ML paradigms—an SVM model based on handcrafted GLCM texture features and a CNN model with end-to-end feature learning—were developed for melting quality classification, and a comprehensive parametric analysis was conducted to evaluate the effects of critical model factors (feature dimensions and kernel functions for SVM, as well as network depth, kernel size, and kernel number for CNN) on classification performance. Finally, the two ML models were comprehensively compared in terms of classification accuracy, misclassification patterns, and inference efficiency to provide practical guidance for model selection in industrial L-PBF in situ monitoring.

The remainder of this paper is organized as follows: Section 2 details the experimental setup, including the L-PBF processing system, in situ layer-wise image monitoring platform, experimental design, and dataset establishment procedure. Section 3 presents the development and parametric optimization of the two ML-based melting quality classification models, followed by a comprehensive comparative evaluation of their performance. Finally, Section 4 summarizes the key findings of this study and outlines prospects for future research on intelligent in situ monitoring and closed-loop control of the L-PBF process.

## 2. Experimental Setup and Data Acquisition

This section details the L-PBF processing system, in situ layer-wise image monitoring platform, experimental design for fabricating specimens with controlled melting states, and the procedure for establishing the high-quality labeled image dataset. The overall experimental workflow is designed to systematically correlate L-PBF process parameters, layer-wise melting quality, and surface image characteristics. This provides a reliable data foundation for subsequent machine learning (ML)-based quality recognition.

### 2.1. L-PBF Processing Platform

All L-PBF forming experiments were conducted on a commercial Farsoon FS121M metal additive manufacturing system (Farsoon Technologies Co., Ltd., Changsha, China), which is widely validated for high-precision metal AM fabrication in industrial and academic research. The system is equipped with a build volume of 120 mm × 120 mm × 100 mm, providing sufficient space for parallel fabrication of multi-group specimens with different process parameters. The core energy source is a single-mode fiber laser with a maximum output power of 500 W and a focused spot diameter of 70 μm, enabling stable and controllable energy input to ensure consistent melting of metal powder.

To prevent high-temperature oxidation of the molten pool and guarantee the metallurgical quality of as-built parts, high-purity argon (99.999%) was used as the protective atmosphere throughout the entire fabrication process. Before printing, the forming chamber was purged with argon to reduce the oxygen content below 100 ppm (0.01%), and this low-oxygen environment was maintained continuously during the build cycle. The laminar argon flow also removes laser-induced fumes and spattered particles in real time, minimizing their adverse effects on the powder bed surface and the melting quality of subsequent layers.

### 2.2. In Situ Layer-Wise Image Monitoring Platform and Image Preprocessing

To capture the layer-wise surface morphology of as-built parts and realize non-intrusive in situ monitoring of the L-PBF process, a machine vision-based monitoring system was integrated into the Farsoon FS121M platform. A high-resolution complementary metal–oxide–semiconductor (CMOS) camera (Basler acA2040-180km, 2048 × 2048-pixel resolution, Basler AG, Ahrensburg, Germany) with a 16 mm fixed-focus industrial lens was mounted outside the sealed observation window of the build chamber, ensuring no interference with the normal equipment operation or powder spreading process. The camera was pre-calibrated using a standard checkerboard calibration plate to eliminate optical distortion, ensuring the geometric accuracy of the captured images.

The image acquisition process was synchronously triggered by the machine’s control system via a programmable logic controller signal switch, ensuring strict time synchronization with the L-PBF process steps. For each layer, an image was captured immediately after the laser scanning of the solid part was completed and before the next layer of powder was spread—this timing ensures the surface morphology of the part (after melting and solidification) is recorded without powder coverage.

A two-step preprocessing pipeline was developed to eliminate interference factors and improve raw image quality:

Perspective correction: A homography transformation-based perspective correction algorithm was applied to convert the tilted view (caused by the off-axis installation angle of the camera) into a standard top-down orthographic view of the part, ensuring consistent perspective across all layer images.

Region of interest (ROI) extraction: An automatic ROI cropping algorithm was developed to segment and extract the target part area from the corrected images, removing irrelevant background information (e.g., surrounding powder bed, build chamber inner wall, substrate). This step not only reduces data volume for subsequent model training and inference but also eliminates background noise interference to improve melting quality recognition accuracy.

### 2.3. Experimental Materials and Design

Gas-atomized spherical 316L austenitic stainless-steel powder (manufactured by Avimetal AM Tech Co., Ltd., Beijing, China) was used as the raw material for all experiments—this material is widely adopted in industrial L-PBF applications due to its excellent corrosion resistance and processability. The powder has a particle size distribution of D10 = 30 μm, D50 = 55 μm, and D90 = 80 μm. Before each forming, the powder was pretreated to ensure processing stability: it was sieved through a 200-mesh standard screen to remove agglomerates and impurities, and then dried in a vacuum drying oven at 120 °C for 2 h to eliminate residual moisture (which avoids pore defects caused by moisture vaporization during laser melting).

To obtain specimens with controllable and distinct melting states (OM, LOF, and normal melting), a series of cubic specimens with a cross-sectional dimension of 20 mm × 20 mm was designed. Laser power and scanning speed were selected as the only variable process parameters, as these two factors dominantly determine the volumetric energy density (VED) input into the powder bed—directly governing the material’s melting state.

The volumetric energy density (VED) [30,31] of each parameter group was calculated using the standard L-PBF process formula:

$$\mathrm{VED}=\frac{P}{v\cdot h\cdot s}$$

where *P* is the laser power (W), *v* is the scanning speed (mm/s), *h* is the layer thickness (mm), and *s* is the scanning spacing (mm). Scanning spacing (70 μm) and layer thickness (30 μm) were fixed throughout all experiments, consistent with the equipment’s industrial default configuration.

**Figure 1:**
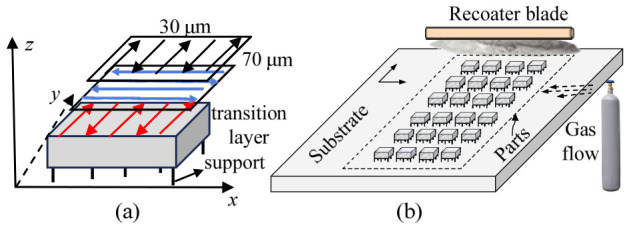
Specimen fabrication strategy and build layout: (**a**) scanning strategy; (**b**) specimen layout and gas flow direction.

A snake-like scanning strategy was adopted for all solid layers, with the scanning direction rotated by 90° between adjacent layers to reduce residual stress accumulation and improve as-built part uniformity. The forming strategy and specimen dimensions are illustrated in Figure 1a. The forming structure of each specimen was divided into three sections:

1. The bottom 100 layers served as the support base, fabricated with the equipment’s recommended process parameters to ensure strong adhesion between the specimen and the substrate, and prevent warpage during the initial build stage.
2. Layers 101 to 121 were transition layers, also fabricated with the standard recommended parameters (laser power: 190 W, scanning speed: 800 mm/s), to provide a stable and uniform surface foundation for the subsequent experimental layers, which eliminated the influence of base layer unevenness on the melting quality of the experimental layers.
3. Layers above 121 were the experimental solid layers, fabricated with the designed variable parameter combinations to induce different melting states.

A total of 24 groups of laser power and scanning speed combinations were designed, covering a wide VED range from 89.3 J/mm^3^ to 294.6 J/mm^3^ to ensure distinct OM, LOF, and normal melting states. The arrangement of specimens on the build platform is shown in Figure 1b. For each build job, 24 cubic specimens were arranged in a 6 × 4 grid on the substrate. The inert gas flows from right to left across the build chamber, as indicated by the arrow in Figure 1b. With this configuration, specimens on the right side are exposed to cleaner gas with minimal spatter, while those on the left may receive some spatter carried by the gas flow. However, this does not affect the validity of the melting quality recognition task. The ground-truth labels (OM, LOF, and normal melting) for each specimen were determined based on VED and confirmed by surface roughness measurements. The specific process parameters and measured surface roughness (Ra) of each specimen are listed in Table 1.

### 2.4. Surface Morphology Characterization and Labeled Dataset Establishment

The surface morphology and melting quality of all fabricated specimens were quantitatively characterized using a laser scanning confocal microscope (Keyence VK-X2000, KEYENCE Corporation, Osaka, Japan), with the arithmetic mean surface roughness (Ra) extracted as the core quantitative evaluation index. It should be noted that there is no universal rigid threshold to separate different melting states, and OM, LOF, and normal melting present continuous transitional characteristics in actual fabrication. Referring to the feasible process window of 316L stainless steel reported in the literature [32], combined with calculated VED, measured roughness data, and microscopic morphology observation, specimens were classified into three melting quality categories. The specific classification criteria are defined as follows:

1. OM: VED > 160 J/mm^3^ and Ra > 10 μm;
2. LOF: VED < 120 J/mm^3^ and Ra > 10 μm;
3. Normal melting: Ra < 10 μm, with uniform and dense surface morphology.

The 10 μm Ra threshold was empirically determined from our experimental data as a practical cutoff separating visually smooth surfaces (normal melting) from rough surfaces (OM/LOF).

**Figure 2:**
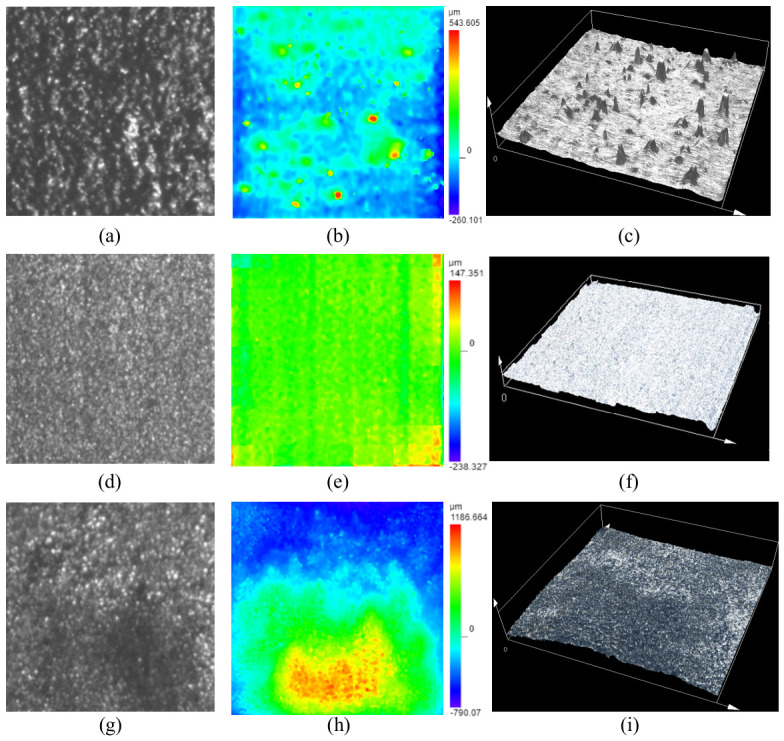
Surface images and microscopic morphologies of three states: (**a**–**c**) surface of the OM part, height of the surface and three-dimensional morphology; (**d**–**f**) surface of the normal melted part, height of the surface and three-dimensional morphology; (**g**–**i**) surface of the LOF part, height of the surface and three-dimensional morphology.

Typical layer-wise surface images and corresponding 3D microscopic morphologies of the three melting states are shown in Figure 2. Distinct image feature differences are observed between categories, providing a solid visual basis for ML-based classification:

OM: Ravine-like surface with strong peak-valley contrast, accompanied by discrete high-brightness spots corresponding to redeposited large spatter particles.

Normal melting: Uniform and fine surface texture with homogeneous grayscale distribution, presenting a smooth metallic luster and no prominent peaks/valleys.

LOF: Cloudy grayscale tone with obvious black granular features, corresponding to incompletely melted powder particles and discontinuous melt tracks.

Combined with the collected layer-wise images, the surface images of parts under different melting qualities exhibit significant differences that reflect the underlying physical mechanisms: (Figure 2a–c) OM state. The surface is characterized by a ravine-like morphology with pronounced peak-to-valley contrast. High-brightness discrete spots are distributed across the image, corresponding to large-sized spheroidized particles formed by molten pool spattering. Microscopically, the melt tracks appear relatively continuous, but isolated tall peaks—attributed to spatter particles—are evident on the surface. (Figure 2d–f) normal melting: The surface displays a uniform and delicate texture with homogeneous pixel gray distribution, presenting a smooth appearance and metallic luster. The microscopic morphology is uniform, with no prominent peaks or valleys, indicating stable melting and solidification. (Figure 2g–i) under-melting: The surface exhibits a cloudy gray tone accompanied by pronounced black granular features. Microscopically, the melt tracks are discontinuous with localized height variations. This morphology results from incomplete melting of powder particles, and the dragging effect of the recoating system during powder spreading leads to local agglomerations or protrusions. The differences in image features between different states provide a reliable basis for online identification and classification of melting quality based on computer vision.

Due to the layer-wise processing nature of L-PBF, layer-wise thermal accumulation and laser energy attenuation caused by fumes may lead to melting quality differences between the outermost and inner layers—potentially causing dataset label inaccuracy. To eliminate this interference, an abnormal layer filtering strategy based on grayscale distribution similarity was adopted.

The histogram similarity degree is measured via the Pearson correlation coefficient of image gray-level distribution features, and the specific calculation formula is shown in Equation (1):

(1) $$C(H_{1},H_{2})=\frac{\sum_{i}\left(H_{1}(i)-{\bar{H}}_{1})(H_{2}(i)-{\bar{H}}_{2}\right)}{\sqrt{\sum_{i}{\left(H_{1}(i)-{\bar{H}}_{1}\right)}^{2}\sum_{i}{\left(H_{2}(i)-{\bar{H}}_{2}\right)}^{2}}}$$

where *H*_1_ and *H*_2_ denote the gray-level distribution features of two images for comparison. The correlation coefficient C ranges from [−1, 1], where a value closer to 1 indicates a higher similarity in gray distribution between the two images, and a value closer to −1 indicates a greater difference.

**Figure 3:**
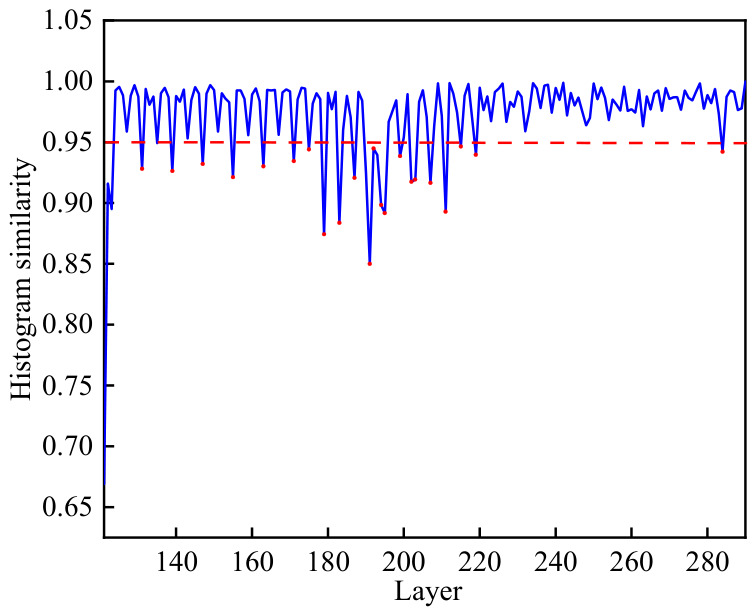
Histogram similarity plot between the inner layer and the outermost layer.

The filtering workflow is as follows:

1. Take the gray-level distribution feature of the outermost layer image of each sample as the benchmark reference;
2. Calculate the distribution similarity between each internal layer image and the benchmark image layer by layer;
3. Set a similarity threshold of 0.95—layers with a similarity coefficient below 0.95 are judged as abnormal and excluded from the dataset (Figure 3).

To further guarantee label accuracy, all filtered layer images underwent two rounds of manual inspection and verification by two independent researchers, with mislabeled images corrected or removed. The final dataset contains 5110 high-quality labeled layer images, including 2410 LOF images, 1140 OM images, and 1560 normal melting images. This dataset was randomly divided into a training set, validation set, and test set at a 7:1:2 ratio for subsequent model training and performance evaluation.

## 3. Construction and Performance Analysis of In Situ Melting Quality Monitoring Model

An in situ melting quality monitoring system is critical for mitigating abnormal melting during the L-PBF process. By intelligently detecting melting quality deviations from surface images captured by the CMOS camera, the system can provide data-driven decision support for process adjustment and adaptive parameter optimization. Essentially, this task can be framed as an image classification problem, which is well-suited for solutions via machine learning (ML) techniques. Two mainstream ML approaches are widely adopted for L-PBF melting quality evaluation: traditional statistical classifiers (e.g., support vector machine, SVM) relying on handcrafted features, and deep learning-based convolutional neural networks (CNNs) capable of end-to-end feature learning from raw images. Both approaches were implemented in this study to explore a robust and efficient method for online melting quality recognition of L-PBF parts.

### 3.1. SVM-Based Melting Quality Classification

#### 3.1.1. Fundamentals of SVM

**Figure 4:**
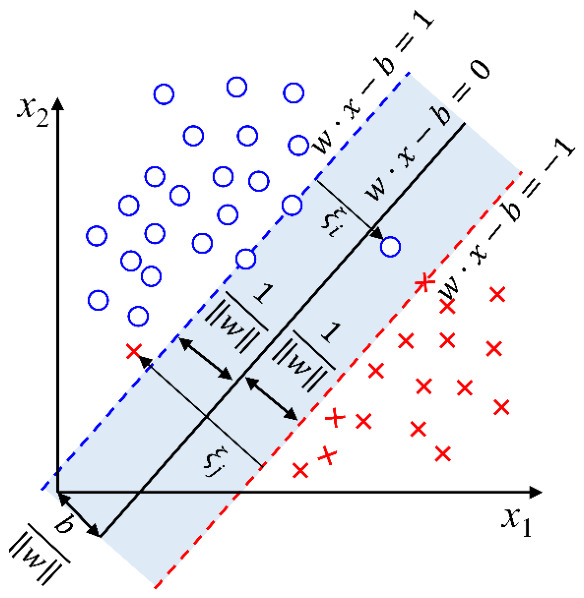
The schematic of an SVM.

SVM aims to find a maximum-margin hyperplane that separates different classes, maximizing the distance between the hyperplane and the nearest samples of each class (Figure 4). This margin maximization strategy enhances the model’s generalization ability.

To handle outliers or noise in the training set, a soft-margin SVM is introduced by incorporating slack variables (ξ_i_), controlled by a penalty factor *C* that balances classification error and hyperplane complexity. For linearly inseparable data, a kernel function maps the feature space to a higher dimension where linear separation is feasible. In this study, the radial basis function (RBF) kernel is adopted:

(2) $$k_{\gamma }(x,x^{\prime })=e^{-\gamma {\left\|x-x^{\prime }\right\|}^{2}}\;,$$

where γ is a hyperparameter controlling the width of the Gaussian distribution—governing the model’s sensitivity to individual samples.

#### 3.1.2. SVM Performance Analysis for Melting Quality Classification

Feature extraction is a prerequisite for SVM-based melting quality evaluation. It is hypothesized that different melting states (OM, LOF, normal melting) can be distinguished by variations in grayscale intensity and texture patterns of surface images. The gray-level co-occurrence matrix (GLCM) [33] is a well-established texture descriptor that records the frequency of pixel value pairs at a fixed distance (d) and direction (θ) in an image. In this study, the gray level was set to 256, and four GLCMs were constructed for directions θ = 0°, 45°, 90°, and 135° (π/4, π/2, 3π/4 radians) with a fixed distance *d* = 1 pixel—ensuring rotation-invariant texture features.

To enhance classification efficiency, statistical features were extracted from the GLCMs, including contrast, energy, correlation, and homogeneity. Each feature yielded four values (one for each direction), and the average value was used to minimize directional bias. Additionally, image entropy and mean local variance were incorporated as supplementary discriminative features. Detailed descriptions of these features are provided in Table 2.

Due to the significant differences in feature scales (e.g., entropy ranges from 0 to 8, while contrast can exceed 10,000), z-score standardization was applied to eliminate dimensionality effects and ensure fair feature contribution during SVM training. The standardization formula is

(3) $$x^{*}=\frac{x-\mu }{\sigma },$$

where *μ* and *σ* are the mean and standard deviation of the training set features, respectively.

SVM training was implemented using the scikit-learn library (version 1.0.2), with five-fold cross-validation to evaluate model performance, mitigating overfitting and ensuring reliable accuracy estimates.

**Figure 5:**
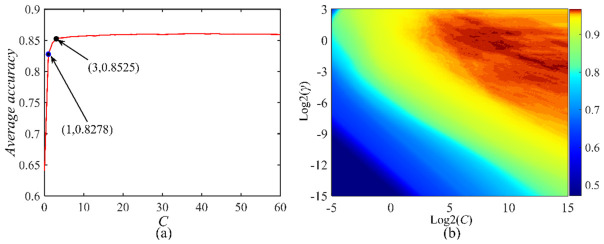
The average accuracy varies with parameters for SVM with (**a**) linear kernel; (**b**) RBF kernel.

The penalty factor *C* plays a critical role in balancing classification accuracy and model generalization. A larger *C* imposes a stricter penalty for misclassification, potentially leading to overfitting, while a smaller C may result in underfitting. Figure 5a illustrates the variation in average classification accuracy with C for the linear kernel SVM. Accuracy increases from 0.7505 (*C* = 0.1) to 0.8525 (*C* = 3) and then stabilizes with further increases in *C*—indicating limited improvement in generalization beyond *C* = 3. Overall, the linear kernel SVM exhibits poor performance for melting state classification, which can be attributed to the nonlinear nature of image texture features.

To address this limitation, the nonlinear RBF kernel was adopted, with performance governed by two hyperparameters: *γ* (kernel width) and *C* (penalty factor). Figure 5b presents a heatmap of classification accuracy as a function of log_2_(*C*) and log_2_(*γ*), where the red “basin” indicates the optimal parameter region. The highest accuracy of 96.77% was achieved with the parameter combination (log_2_*C* = 12.4, log_2_*γ* = −2), representing a 10% improvement over the linear kernel SVM.

**Figure 6:**
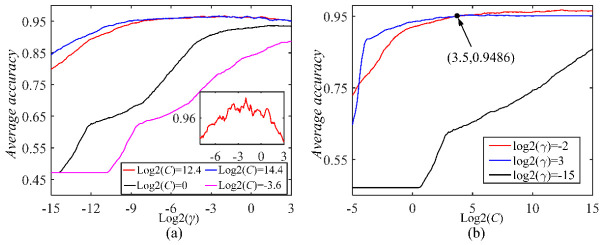
The accuracy curve varies with parameters (**a**) γ and (**b**) C.

Figure 6a,b depict the accuracy trends as a function of *γ* (for fixed *C* values) and *C* (for fixed *γ* values), respectively. For smaller *C* values (more tolerant to misclassification), accuracy increases monotonically with *γ* (black and pink lines in Figure 6a). In contrast, for larger *C* values (stricter misclassification penalty), accuracy first increases and then decreases with *γ* (red and blue lines)—indicating overfitting at high *γ*. Similarly, Figure 6b shows that accuracy generally increases with *C*, but the upper limit is constrained by small *γ* values (black line). Beyond the intersection point (*C* = 3.5, accuracy = 0.9486), the model with a smaller *γ* (smoother decision surface) outperforms the larger *γ* counterpart—highlighting the trade-off between model complexity and generalization.

**Figure 7:**
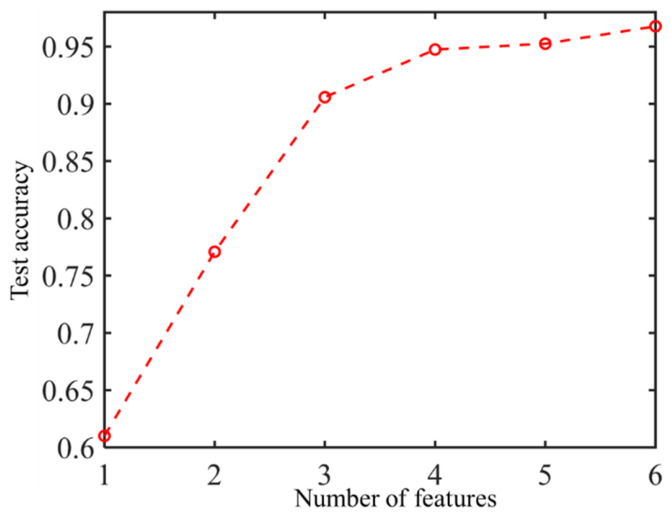
Accuracy varies with the number of features.

The number of features also influences SVM performance. Figure 7 illustrates accuracy as a function of feature count under the optimal parameter combination (log_2_*C* = 12.4, log_2_*γ* = −2). Accuracy increases with the number of features, reaching 0.9058 with 3 features and peaking at 96.77% with all 6 features. Thus, all extracted features were retained for subsequent SVM modeling.

### 3.2. CNN-Based Melting Quality Classification

#### 3.2.1. Fundamentals of CNN

**Figure 8:**
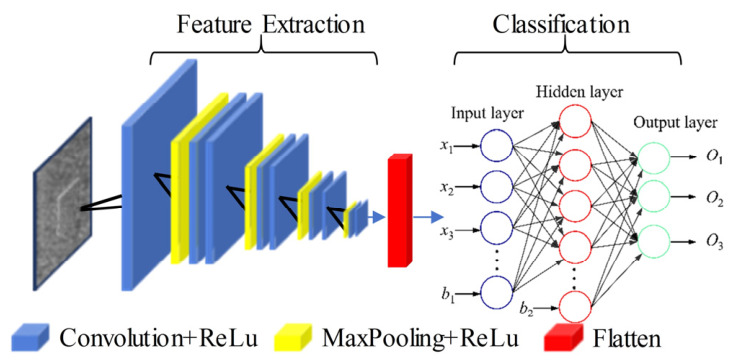
CNN-based melting state recognition.

CNN is a widely used deep learning architecture for image classification, capable of automatic feature extraction from raw images, eliminating the need for manual feature engineering (a major limitation of SVM). As shown in Figure 8, a typical CNN consists of stacked convolutional layers, pooling layers, and fully connected (FC) layers—forming a hierarchical feature-learning framework.

The convolutional layer (Conv) serves as the core feature extractor, utilizing learnable kernels (filters) that slide across the input image to generate feature maps. The output of the convolution operation is given by:

(4) $$O_{n}^{j}=f(\sum_{i\in M}O_{i}^{j-1}*W_{k\in n}^{j}+b_{n}^{j}),$$

where *O*_*n*,*j*_ denotes the *n*-th feature map from the *j*-th layer; *f*(·) is the nonlinear activation function (ReLU was adopted to mitigate the vanishing gradient problem); *M* is the number of input channels; * represents the 2D convolution operation; *W*_*n*,*j*_, *k* is the weight matrix of the *n*-th kernel in the *j*-th layer (shared across the entire image); *I_k_* is the *k*-th input feature map; and *b*_*n*,*j*_ is the bias term for the *n*-th feature map in the *j*-th layer.

Following convolution, a pooling layer is introduced to reduce the spatial dimension of feature maps—lowering computational complexity and enhancing model generalization. Max-pooling (MaxP) was employed in this study, which selects the maximum value within a fixed local receptive field (e.g., 2 × 2 pixels) to downsample feature maps while preserving critical edge and texture information. A “convolutional module” is defined as one convolutional layer followed by one pooling layer.

Subsequently, the extracted feature maps are flattened into a 1D feature vector and fed into the FC layers—functioning as a multilayer perceptron (MLP) that maps high-dimensional features to target classification labels. The final layer employs a softmax activation function to convert FC layer outputs into class probabilities, representing the likelihood of the input image belonging to each melting state (OM, LOF, normal melting). The softmax function is defined as

(5) $$p_{i}=\frac{e^{y_{i}}}{\sum_{j}^{n}e^{y_{j}}},$$

where *p_i_* is the probability of the input belonging to the *i*-th class; *y_i_* is the FC layer output for the *i*-th class; and n is the number of classification classes (*n* = 3 in this study).

#### 3.2.2. CNN Performance Analysis for Melting Quality Classification

CNN performance is highly dependent on architectural design, including network depth (number of convolutional modules), kernel size, number of kernels per convolutional layer, and the structure of the FC layers. Currently, no standardized theoretical framework exists for optimizing these parameters, and architectural selection relies primarily on empirical investigation and iterative refinement.

All CNN experiments were conducted on a Windows 10 workstation equipped with an Intel Xeon Silver 4210R CPU and an NVIDIA GeForce RTX 3090 GPU, accelerating model training via parallel computing. Models were implemented using the PyTorch library (PyTorch 1.12.1 with Python 3.8), with cross-entropy loss as the objective function and the Adam optimizer (PyTorch 1.12.1 with Python 3.8) for parameter updates. The learning rate and batch size were fixed at 0.0001 and 32, respectively—determined via preliminary experiments to balance training stability and convergence speed.

To systematically investigate the influence of architectural parameters, a consistent notation was adopted:

- D-*i*: Convolutional depth (*i* convolutional modules);
- S-*j*: Convolutional kernel size (*j* × *j* pixels);
- N-*z*: Number of kernels per convolutional layer (*z* kernels).

Under this notation, D-0 represents a degenerate configuration (no convolutional modules), reducing the network to an MLP operating directly on flattened image pixels. Although a single-hidden-layer MLP can theoretically approximate any nonlinear function, practical constraints (e.g., limited training data, computational burden) restrict its performance—especially for high-dimensional image data.

**Figure 9:**
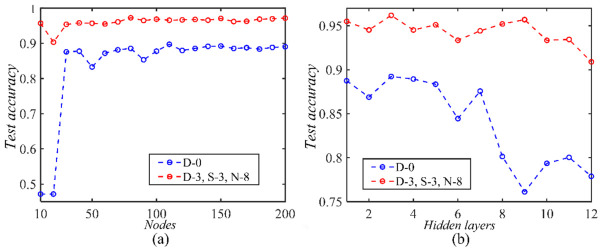
The test accuracy varies with (**a**) the number of nodes in the hidden layer and (**b**) the depth of the hidden layer, with 30 nodes fixed at each layer.

Figure 9a compares the test accuracy of MLP (D-0) and CNN (D-3) architectures as a function of hidden layer node count. For the MLP (blue circles), accuracy remains low (≤0.8) with 10–20 nodes, improves to 0.8457 with 30 nodes, and fluctuates between 0.83 and 0.89 with further node increases—indicating diminishing returns and potential overfitting. In contrast, the CNN (red circles) achieves an accuracy of 0.9569 even with 10 hidden layer nodes, and accuracy remains stable with additional nodes. Overall, incorporating convolutional layers yields an ~8% accuracy improvement compared to the pure MLP—validating the effectiveness of automatic feature learning for melting quality classification.

Figure 9b illustrates the effect of hidden layer depth (fixed at 30 nodes per layer) on test accuracy for MLP and CNN (D-3). For both models, accuracy decreases with increasing hidden layer depth—attributed to overfitting and the vanishing gradient problem in deep networks. However, the CNN maintains significantly higher accuracy than the MLP across all depths, further confirming its superiority for image-based melting quality recognition.

**Figure 10:**
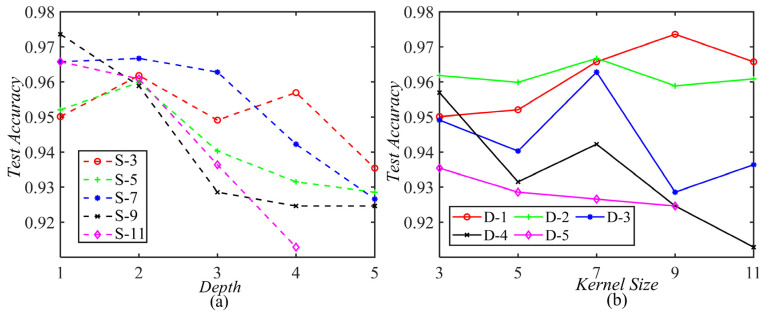
Test accuracy varies with (**a**) the depth of convolutional modules and (**b**) the kernel size.

To balance accuracy and computational efficiency, the FC module was fixed as a single hidden layer with 30 nodes for subsequent experiments. The influence of convolutional module depth (D-*i*) and kernel size (S-*j*) on test accuracy is shown in Figure 10a,b. As depicted in Figure 10a, excessive convolutional depth leads to accuracy degradation regardless of kernel size—due to cumulative information loss from multi-stage pooling, which impairs the model’s ability to capture fine-grained melting state features. Notably, the network fails to converge (accuracy drops sharply) when using 11 × 11 kernels (pink dotted line) with 5 convolutional modules, resulting from excessive spatial dimension reduction. The highest accuracy of 0.9735 is achieved with a single convolutional module (D-1) and 9 × 9 kernels.

From the perspective of kernel size (Figure 10b), the trend in accuracy varies with convolutional depth:

- For deep networks (D = 4, 5; black and pink solid lines), accuracy decreases with increasing kernel size—large kernels combined with deep modules cause over-sampling of features and information loss;
- For a single convolutional module (D = 1; red solid line), accuracy increases with kernel size—larger kernels capture more global texture features of the part surface;
- For two convolutional modules (D = 2; green solid line), accuracy fluctuates slightly with kernel size, indicating a balance between local and global feature extraction.

**Figure 11:**
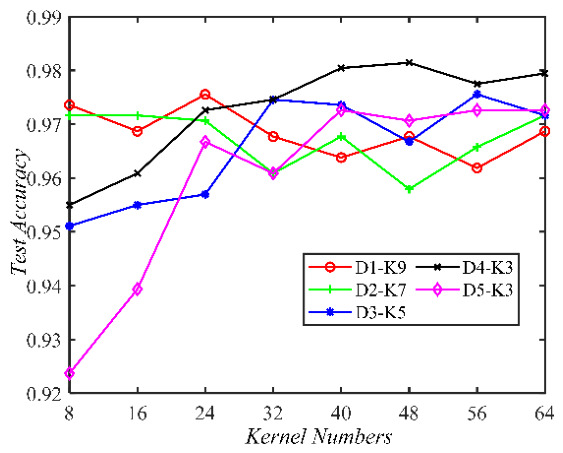
The accuracy curve varies with the number of kernels.

After selecting the optimal kernel size for each convolutional depth (based on Figure 10b), the influence of the number of kernels per convolutional layer (N-*z*) on test accuracy was evaluated (Figure 11). Models with large kernels and shallow depth (red and green dotted lines) exhibit decreasing accuracy with increasing kernel count—likely due to overfitting from feature redundancy. In contrast, models with small kernels (3 × 3) and deeper depth (D = 4, 5; black and pink dotted lines) show increasing accuracy with more kernels—additional kernels enable richer feature extraction without significant information loss. The highest test accuracy of 98.14% is achieved with the optimal CNN architecture: 4 convolutional modules (D-4), 3 × 3 kernels (S-3), and 48 kernels per convolutional layer (N-48).

### 3.3. Comparative Evaluation of SVM and CNN Classifiers

While the preceding analysis demonstrates that CNN achieves higher overall accuracy than SVM, accuracy alone is insufficient to evaluate classifier performance—especially for multi-class problems with imbalanced class distributions. A classifier could achieve high accuracy by biasing toward the majority class while failing to recognize minority classes, which is unacceptable for industrial applications where reliable identification of all melting states (OM, LOF, normal) is critical.

**Figure 12:**
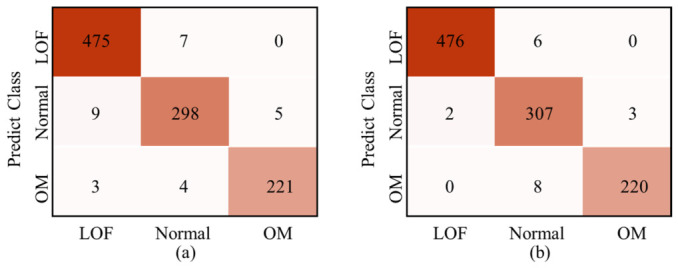
Confusion matrices with (**a**) SVM and (**b**) CNN.

To provide a comprehensive assessment, confusion matrices and key performance metrics (precision, recall, F1-score) were analyzed for both classifiers. Figure 12 presents the confusion matrices for SVM and CNN on the test dataset, and Table 3 summarizes the quantitative performance indicators.

The results reveal several critical distinctions between the two classifiers:

Accuracy and reliability: CNN outperforms SVM in both precision and recall for LOF and normal melting states, indicating more robust identification of these critical melting conditions. For OM, CNN exhibits slightly lower precision (0.9649 vs. 0.9736 for SVM) but higher recall (0.9865 vs. 0.9693 for SVM), suggesting a marginal tendency to misclassify non-OM samples as OM, but better detection of actual OM defects.

Misclassification patterns: A qualitative analysis of the confusion matrices (Figure 12) reveals a critical flaw in the SVM: occasional misclassification of OM samples as LOF (off-diagonal entry in Figure 12a). This type of cross-class misclassification has severe industrial consequences; misidentifying OM (excessive energy) as LOF (insufficient energy) would prompt operators to increase laser power, exacerbating defects and accelerating process degradation. In contrast, CNN exhibits no such misclassifications (Figure 12b), avoiding catastrophic operational errors and enhancing decision support reliability.

Computational efficiency: Both classifiers meet real-time monitoring requirements (inference time < 0.1 s/layer), but CNN achieves a significantly faster inference speed (0.036 s/layer) compared to SVM (0.068 s/layer)—nearly halving the latency. This efficiency advantage stems from the CNN’s end-to-end architecture, which eliminates the separate feature extraction step required for SVM.

In summary, while both SVM and CNN demonstrate viable performance for melting quality recognition, CNN offers three key advantages for practical industrial deployment: higher overall accuracy, elimination of critical cross-class misclassifications between physically opposite melting states, and superior computational efficiency. These characteristics position CNN as a more robust and reliable foundation for operator decision support in L-PBF in situ monitoring.

## 4. Conclusions

This study systematically investigated machine learning-based layer-wise melting quality recognition for L-PBF in situ monitoring. Two models—SVM with GLCM texture features and an optimized CNN—were developed, evaluated, and compared using a high-quality dataset of 5110 layer-wise images covering three melting states: OM, LOF, and normal melting.

The optimized CNN achieved a classification accuracy of 98.14%, outperforming the SVM (96.77%) by 1.37%. More importantly, the CNN eliminated the fatal cross-misclassification between OM and LOF observed in the SVM—an error that would trigger catastrophic process adjustments in real-world deployment. The CNN also demonstrated significantly faster inference speed (0.036 ± 0.004 s/layer) compared to the SVM (0.068 ± 0.004 s/layer), meeting the real-time requirements of L-PBF in situ monitoring.

These results establish the CNN as the preferred approach for practical industrial deployment, offering robust detection of both under-energy and over-energy defects while maintaining computational efficiency. Future work will focus on online implementation of the CNN model, integration with multi-sensor data fusion (e.g., melt-pool thermal imaging), and generalization to other metal materials and L-PBF systems.

## Figures and Tables

**Table 1:** Designed L-PBF process parameters and corresponding surface roughness of as-built specimens.

Specimen ID	Scanning Speed (mm/s)	Laser Power (W)	Ra (μm)	Specimen ID	Scanning Speed (mm/s)	Laser Power (W)	Ra (μm)
1	400	120	21.85	14	400	300	10.39
2		150	28.64	15	600		25.16
3		180	35.08	16	800		26.83
4		210	13.90	17	1000		15.36
5		240	11.81	18	1200		29.36
6		270	12.82	19	400	210	13.90
7		300	10.39	20	500		35.44
8		330	8.37	21	600		33.36
9	700	180	33.98	22	700		17.75
10		210	17.75	23	800		19.34
11		240	25.40	24	900		20.61
12		270	29.95				
13		300	38.77				

**Table 2:** Feature description used for SVM classification.

Feature	Mathematical Expression	Description
Contrast	$\sum_{i,j}p_{i,j}{\left(i-j\right)}^{2}$	Reflects the intensity difference between a pixel and its neighbors
Energy	$\sum_{i,j}{\left(p_{i,j}\right)}^{2}$	Represents the orderliness and texture coarseness
Correlation	$\sum_{i,j}\frac{(i-\mu_{i})(j-\mu_{j})p_{i,j}}{\sigma_{i}\sigma_{j}}$	Captures linear dependencies between neighboring pixels
Homogeneity	$\sum_{i,j}\frac{p_{i,j}}{{\left(i-j\right)}^{2}}$	Indicates the uniformity of local grayscale distribution
Mean local variance	$\frac{1}{n}\sum_{k=1}^{n}\frac{\sum {\left(X-\mu_{k}\right)}^{2}}{N}$	Quantifies local intensity fluctuations across the image
Entropy	$-\sum_{i=0}^{255}\log_{2}p_{i}$	The randomness or complexity of the grayscale distribution

Note: *P*(*i*,*j*) is the probability of pixel pair (*i*,*j*) in the GLCM; *μ_i_*, *μ_j_*, *σ_i_*, *σ_j_* are the means and standard deviations of the GLCM row and column sums; *I*(*x*,*y*) is the grayscale value at pixel (*x*,*y*); *μ_lo_c_al_* is the local mean grayscale value; *M* and *N* are the image height and width, respectively.

**Table 3:** The performance metrics for SVM and CNN classifiers.

Class	CNN		SVM	
	Precision	Recall	Precision	Recall
LOF	0.9876	0.9958	0.9774	0.9855
Normal	0.9840	0.9564	0.9644	0.9551
OM	0.9649	0.9865	0.9736	0.9693
Overall accuracy	0.9814		0.9677	
Inference time (s/layer)	0.036 ± 0.004		0.068 ± 0.004	

## Data Availability

The original contributions presented in this study are included in the article. Further inquiries can be directed to the corresponding author.
